# The endocannabinoid system in zebrafish and its potential to study the effects of Cannabis in humans

**DOI:** 10.1186/s42826-022-00116-5

**Published:** 2022-02-22

**Authors:** Ricardo Lacava Bailone, Hirla Costa Silva Fukushima, Luis Kluwe de Aguiar, Ricardo Carneiro Borra

**Affiliations:** 1 Department of Genetic and Evolution, Federal University of São Carlos, Washington Luiz Road, km 230, São Carlos City, São Paulo County 13565-905 Brazil; 2 Department of Federal Inspection Service, Federal Inspection Service, Ministry of Agriculture, Livestock and Supply of Brazil, Brasília, Brazil; 3 Centre of Biological and Health Sciences, Federal University of Sao Carlos, São Carlos, Brazil; 4 Department of Food, Land and Agribusiness Management, Harper Adams University, Newport, England

**Keywords:** 3R, ∆9-tetrahydrocannabinol, Animal model research, Cannabidiol, CB1, CB2

## Abstract

Zebrafish is considered an unprecedented animal model in drug discovery. A review of the literature presents highlights and elucidates the biological effects of chemical components found in *Cannabis sativa.* Particular attention is paid to endocannabinoid system (eCB) and its main receptors (CB1 and CB2). The zebrafish model is a promising one for the study of cannabinoids because of the many similarities to the human system. Despite the recent advances on the eCB system, there is still the need to elucidate some of the interactions and, thus, the zebrafish model can be used for that purpose as it respects the 3Rs concept and reduced time and costs. In view of the relevance of cannabinoids in the treatment and prevention of diseases, as well as the importance of the zebrafish animal model in elucidating the biological effects of new drugs, the aim of this study was to bring to light information on the use of the zebrafish animal model in testing *C. sativa-*based medicines*.*

## Background

Belonging to the *Cannabaceae* family, *Cannabis sativa* is a plant with more than 500 active chemical compounds already isolated, among which about 17% of these are classified as cannabinoids. These are widely used in different preparations and formulations for both medical and recreational purposes as they can decrease the level of stress, anxiety, and depression [1–3]. Studies in animal models have shown that cannabidiol (CBD) can be used to treat a variety of diseases such as autism, fibromyalgia, multiple sclerosis, Alzheimer's, Parkinson's, epilepsy and seizures, schizophrenia, and psychosis [4–12], among others. Due to its anti-proliferative, pro-apoptotic and cell migration inhibition activities, CBD has also been highly used in the treatment of tumors, gaining even greater prominence for its medicinal use in each animal and human health [13]. Further to CBD, which is a non-psychotomimetic compound [14], ∆9-tetrahydrocannabinol (THC) [15, 16] is a psychoactive compound and one of the primary active constituents of cannabis [17]. Both CBD and THC can be either produced synthetically, or they can be extracted from the Cannabis plant as an essential oil [18].

Due to the increased use of cannabinoids in medical treatment, it is deemed of great relevance to look at toxicity studies of the components of Cannabis to ascertain the patients’ safety. Currently, information on the precise mechanisms of action is limited. Thus, to achieve a more accurate understanding of the pharmacological effects of this plant in the body, it might be essential to perform laboratory research the use of animal models [19–21]. Consequently, a high-throughput in vivo model to understand the connection between the chemical composition of different strains and their therapeutic potential then turns into of value. This would then justify the use of the zebrafish model as an alternative [22].

Usually, for the purpose of studies of drugs, murine species, mainly mice and rats are used to test their effects. However, the zebrafish model (*Danio rerio*) has presented itself to be a promising alternative. Firstly, it respects the principle of 3Rs (reduction, replacement, and refinement) [23], and due to the rapid embryonic development, studies can be conducted using embryos and larvae up to 120 h post fertilization (hpf).

According to international animal welfare ethical regulations [24, 25], the use of the zebrafish model can be considered an in vitro model. Furthermore, the transparency of embryos and larvae allows the drug's effect to be evaluated in vivo and in real time under an optical microscope. Due to genetic homology and external fertilization, zebrafish knockout models for human diseases have been easily developed, such as for epilepsy models developed for the study of Dravet's syndrome [26]. Thus, innovative treatments using CBD and THC can be evaluated quickly and effectively, and with a high degree of specificity.

In addition, zebrafish’s unique ADME (absorption, metabolism, and excretion) system during the first days of its life, could provide invaluable insights into the mechanisms of toxicity of plants’ components used for medicinal purposes which would hopefully help to identify and discover new compounds for future treatments [27]. Studies with zebrafish range from evaluating the toxicity of bioactive compounds or crude plant extracts to determining their process of action and optimal dosage for human diseases. Due to its specific experimental advantages that facilitate a large-scale phenotypic approach, toxic changes in the neural, cardiac, hepatic, and immune systems can be evaluated with the exposure of zebrafish embryos and larvae to the compounds, whose safety and efficacy results can be obtained in maximum 5 days in 96-well plates.

Studies with the zebrafish animal model and cannabinoids have been going on for some time. In 1974, a study entitled “The toxicologic and teratologic effects of THC in the zebrafish embryo” [28], tested doses between 1.0 to 10.0 ppm in a fish tank. It was noted that there was no cytotoxicity or significant loss of embryos after exposure for 24 h for any tested concentration. At doses above 2 ppm, there was a reduction in spontaneous tail muscle contraction and subsequent embryos death, while at the 2 ppm dose there was no effect on spontaneous muscle contraction or any subsequent embryos death, but 37% of distal trunk anomalies were found, such as curved spine or bulbous-tipped tail. At the end of this experiment, no teratogenic effects or embryonic death had been found at concentrations underneath 2 ppm.

In addition to all these advantages presented above, by the mid-1990s the endocannabinoid system (eCB) which had been identified in humans was also discovered, to be present in zebrafish. This system is composed of endogenous lipid-based retrograde neurotransmitters that bind to cannabinoid receptors, and cannabinoid receptor proteins that are expressed throughout a vertebrate’s central nervous system (including the brain) and peripheral nervous system. Despite the preliminary studies on the eCB system so far, it is still certain what is the mechanism responsible for regulating and balancing other systems in vertebrates such as physiological, cognitive processes, fertility and pregnancy, pre- and postnatal development, immunology, appetite, pain sensation, mood, and memory, as well as in the perception of the pharmacological effects of Cannabis [29–34].

In view of the relevance of cannabinoids in the treatment and prevention of diseases, as well as the significance of the zebrafish animal model in elucidating the biological effects of new drugs, the intention of this study is to provide information on the use of the zebrafish animal model based on studies using cannabis*.*

## Main text

A systematic literature review was carried out using databases such as PubMed, Science Direct, Google Scholar, and SciELO (Scientific Electronic Library Online). Emphasis was given on identifying publications using search words and terms containing ‘*Cannabis sativa*’ and ‘Zebrafish’. Particularly, the main keywords searched included ‘Zebrafish model’, ‘Cannabinoid’, ‘Cannabidiol’, ‘Tetrahydrocannabinol’, ‘CBD’, ‘THC’, ‘Diseases’ and ‘Toxicology’. Initially, 122 publications were identified which included books, rulings and articles published by international scientific journals of high impact factor. The publications were selected according to relevance and timeliness, being around 19.5% of the articles used published in the last year, 52% in the last five years and 75% published in the last ten years.

The high genetic, anatomical, and physiological similarity of the zebrafish model to humans has allowed for the replacing of superior vertebrates such as mice, guinea pigs, rats, rabbits, and monkeys which are used in research that require in vivo models to elucidate events. The zebrafish model has been extensively used for modulation of human and animal diseases, behavioral assessment, production of new vaccines, toxicological testing of ingredients and food additives, efficiency of new drugs, among many other tests performed with other animal models, at a lower cost and in less time [23, 35–44]. Below, the physiological similarity of zebrafish to the human endocannabinoid system is discussed in the light of discoveries in the late 1990s [45] and how that animal model could help in the development of innovative treatments and new drugs.

### Endocannabinoid system in Zebrafish

The term 'endocannabinoid' emerged in the mid-1990s, after the discovery of membrane receptors for the psychoactive principle of Cannabis and its components. Endocannabinoid currently indicates a whole signaling system that comprises cannabinoid receptors, endogenous ligands and enzymes for ligand biosynthesis and inactivation. Soon after the discovery of eCB, studies showed that it was involved as a palliative for many diseases and syndromes, showing itself as a trend towards new therapeutic agents [46].

The eCB acts as the communication system between the brain and body processes, and its main function is to help maintain homeostasis. Its presence is widespread throughout the animal kingdom, for that reason found in each of the vertebrate species. Proper regulation of homeostasis is essential to ensure the body's proper functioning, so all other body systems must carefully control the functioning of their cells. Therefore, the eCB is ubiquitous in the body, in cell membranes of the brain, organs, connective tissues, glands and immune system cells. Furthermore, eCB is also found at the intersections of several other systems, allowing for communication and coordination between different cells in the body [47–49].

Several physiological mechanisms occur in the body when cannabinoid receptors are stimulated, such as reduced pain and inflammation, increased appetite, thermoregulation, intraocular pressure, energy balance, metabolism, sleep improvement, stress reduction, motivation, disposition, memory, among others. Cannabinoid receptors’ main components consist of: (i) the receptors per se found on the surface of cells, which transmit information to deeper cells about changes in conditions, initiating an appropriate cellular response; (ii) endocannabinoids, characterized by small cannabinoid receptor activating molecules; (iii) metabolic enzymes that work by breaking down endocannabinoids after their use, so that they are used only when necessary, never longer [47–49].

This is an interesting fact of the eCB system, which acts only on demand, that is, it activates only when necessary and works to repair or modulate the function of other mediators. Evidently, because of their large quantity in the central nervous system (CNS), neurotransmitters are the main candidates for their interaction [46, 50].

**Fig. 1:**
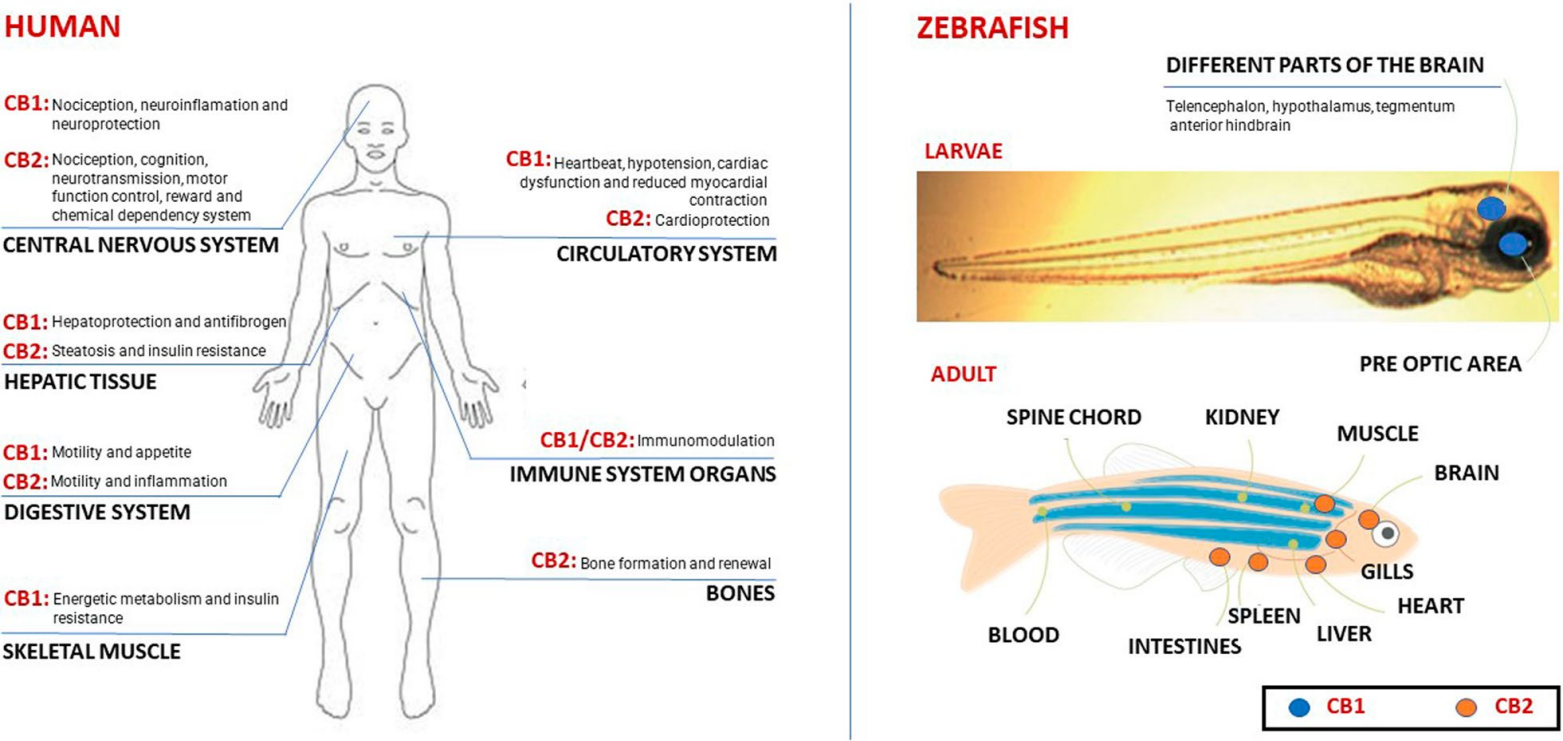
CB1 and CB2 receptors in human and zebrafish (Adapted from Active Pharmaceutica [53])

Within the eCB system, the two main receptors are the cannabinoid receptor 1 (CB1) and the cannabinoid receptor 2 (CB2), and while CB1 receptors are found in the CNS, CB2 receptors are found in the peripheral nervous system. Despite them not being the only ones, they were the first discovered and remain the most studied. Further to CB1 receptors being most abundant in the cerebral cortex, hippocampus, hypothalamus, cerebellum, spinal cord, dorsal cord ganglia, they are also found in the enteric nervous system, adipocytes, endothelial cells, hepatocytes, muscle, and gastrointestinal system. Conversely, CB2 receptors are found in the immune system as part of T and B cells, spleen, tonsils, and activated microglial cells [51] (Fig. 1).

In zebrafish, the expression of zebrafish CB1 was first detected in the pre-optic area as early as day one post-fertilization (dpf). At the later larval stage, CB1 expression was detected in different elements of the brain, which include the telencephalon, hypothalamus, tegmentum, and anterior hindbrain. In adults, CB2 mRNA was detected in gills, heart, intestine, muscle, spleen, and central nervous system [52] (Fig. 1).

### Immunological system × endocannabinoid system

The immune system exists to contain or prevent possible invaders which could potentially affect health. It also acts in preventing the proliferation of cancer cells, fighting bacteria, viruses, and fungi infection. Yet, the immune system can also attack, for example, transplanted organs. Therefore, it is vital that the immune system be able to distinguish what is and is not foreign to the organism, otherwise it can identify antigens as dangerous, and trigger an immune response [54–57].

Although there lack more conclusive studies on how the immune system act, it is known that the connection between the immune system and the endocannabinoid one is mediated by CB1 and CB2. Endocannabinoids and cannabinoids can help regulate the immune response, thus helping to control or neutralize symptoms caused by autoimmune diseases. It can help both HIV-positive people to minimize the symptoms caused by HIV infection and people with multiple sclerosis, one of the most recurrent autoimmune diseases [54–57].

As for the development of tumors, there is growing evidence of that most members of the endocannabinoid system of ligands, receptors and enzymes exert significant effects on the growth, motility, invasion, dissemination, and colonization of tumor cells in distant organs [54–57].

It is noteworthy that, as stated, the cells of the immune system express both CB1 and CB2 and, among them, B lymphocytes. Furthermore, NK cells and monocytes express the highest levels of CB2, which initially suggested that these receptors must play an important role in the function of immune cells [58]. For example, CB2 receptors located on the cells of the immune system appear to play an active role in modulating migration and release of cytokines by immune cells [59–61]. Unlikely CB1, which is mainly expressed in the CNS, as discussed above, CB2 is predominantly expressed in immune cells and appears to have no role in the psychotropic effects of THC, although it binds to both CB1 and CB2 [55, 58, 62, 63].

Even within the immune system, several studies indicate that eCB provides an attractive target for the treatment of neuroinflammation of origin microglial, being able to regulate many aspects of the inflammatory response in the brain via both CB1 and CB2 receptors, acting as immune modulators in the CNS [64].

Dotsey et al. [58] also emphasizes that the "on-demand" activation of eCB signaling during immunization acts as a specific "brake system" to impede evolution and excessive inflammation during immune activation. However, the signaling it also ends up suppressing the natural response to vaccination and may decrease the immune response in general. This can have great relevance in the elderly, where due to age, there is an immunological decline in both the innate and adaptive immune systems, decreasing the capacity of specific response to antigens. Thus, eCB plays an important role in the immune response, especially in immunomodulation, and after stimulation of CB2 by its ligands, it has a potent immunomodulatory role and anti-inflammatory properties.

### Neurological system × endocannabinoid system

Regarding the neurological system, the activation of cannabinoid receptors affects the actions of several neurotransmitters such as acetylcholine, dopamine, GABA, glutamate, serotonin, norepinephrine, and endogenous opioids under normal physiological circumstances [65]. The psychotropic effects of THC are mediated through CB1 receptors, and these effects are limiting its clinical use [66]. CBD does not activate CB1 receptors, as it does not contain psychoactive effects, in addition to exhibiting a wide range of therapeutic properties [67].

As stated earlier, CB1 receptor plays an elementary role in the development of the CNS [68]. Migliarini and Carnevali [69] demonstrated that CB1 receptor is present in zebrafish embryos, and when testing the use of an antagonist CB1, it affected the embryo’s development. Humans and zebrafish have comparable endocannabinoid pathways that each contain CB1 and CB2 receptors, showing similarity between species [70].

However, zebrafish has been considered an appropriate tool for the study of Cannabis signaling due to the fact eCB system was found to be extensively preserved amongst zebrafish and mammals [71]. Importantly, the CB1 receptor is well conserved throughout evolution, as 97% of mouse CB1, 84% of amphibian CB1 and 72% of fish CB1 are identical to the human CB1 [72]. CB1 [69, 73] and CB2 [52, 74] receptors, as well as some endocannabinoid key enzymes, such as fatty acid amide hydrolase, prostaglandin-endoperoxide synthase 2, transient receptor potential Cation Channel 1A, and peroxisome proliferator activated receptor alpha have homologous functions and high nucleotide sequence homology when compared to corresponding human genes [71]. Unlike invertebrates, the eCB system of zebrafish contain orthologs of all human cannabinoid signaling genes except N-acylethanolamine acid amidase gene [70, 75, 76]. Examining the expression in adult zebrafish brain, it showed a similar expression pattern [73, 76–78]. Oltrabella et al. [76] detected a high level of CB2 mRNA expression as early as 4 hpf. The expression was reduced by 12 hpf and then up again. The mRNAs encoding DAGLA and DAGLB (enzymes involved in the biosynthesis of the most abundant endocannabinoid 2-AG) were identified for the first time in the initial cleavage period, indicating that these mRNAs were passed on by the mother [76].

Although exposure to THC did not alter zebrafish behavior in the conditioned place preference task [79], fish acutely exposed to THC exhibit activation of extracellular signal-regulated kinases signaling in the lateral pallium associated with deficits in spatial memory performance [80]. The zebrafish eCB system seems to act by modulating associative learning and memory, in which the stimulation of the CB1 receptor may play a specific role in acquisition and storage of aversive learning and memory, while CB1 blockade enhances cognitive functions [81]. Besides, CB1 activation by THC has been found to restrain acquisition of fear learning, perhaps through impairing stimulus encoding processes in the zebrafish brain pallial area [82]. Like rodents, acute THC exposure causes hypolocomotion in zebrafish [83, 84], while high THC concentrations impair locomotor activity of zebrafish larvae [85]. Treatments with both THC and CBD modify synaptic activity at neuromuscular junctions. Fluorescent labeling of primary and secondary motor neurons revealed a change in branching patterns and a reduction in the number of axonal branches in the trunk musculature, resulting in reduced heart rates, axial malformations, and shorter trunks in zebrafish embryos [86]. CBD exposure at blastula increases developmental dysmorphologies, especially jaw malformation [87]. The double mutant zebrafish CB1 and CB2 had impaired liver development and liver function. It was observed that the inhibition of CB receptor activity compromised liver development and metabolic function, affecting liver differentiation and liver size due to fewer hepatocytes, as well as decreased liver-specific gene expression and cell proliferation [88].

Moreover, both endocannabinoid system and retinoic acid signaling pathways influence lipid deposition during zebrafish embryogenesis, with additive function in lipid abundance during development. These results support zebrafish as a helpful model to estimate the neurobehavioral mechanisms of cannabinoids, as well as the potential involvement of eCB system in regulating different biochemical pathways. However, more studies about the circuit mechanisms underlying eCB role in reward, addiction, and anxiety are needed [89].

According to Oltrabella et al. [76], the manipulation and analysis of eCB in zebrafish through the creation of knockout models could serve to elucidate the effects of cannabinoids. In knockout models where genome inactivation or deletion of a gene has taken place could contribute to a greater understanding of their acute and chronic adverse effects. Knockout models are also essential to research the role of genes that have been sequenced but whose functions have not yet been determined, because by causing a specific gene to be inactivated, and observing differences relative to regular behavior or physiology, researchers can assume its likely role. There are already several zebrafish knockout models, for example to study leukaemia, Down syndrome, diabetes, Vici syndrome, cardiovascular diseases, kidney diseases, among many others [90–96].

### Studies involving cannabinoids and zebrafish

Although the human behavior response can never be completely replicated using zebrafish, the fish model’s experiments suggest that many drug-induced human and zebrafish phenotypes share common genetic and physiological factors [97]. The trend in the use of this animal model in the pharmacology and toxicology of medicines is already recognized by its genetic similarity with other animal models and with other species, as well as humans [98, 99]. And in studies with cannabinoids, it is no different.

Akhtar et al. [85] studied the developmental effects of cannabinoids on zebrafish larvae, and their findings, show that 96 h of exposure in zebrafish embryos starting at 24 hpf can be used to research the teratology of sublethal concentrations of cannabinoids. They proved that in acute exposure, the findings were like the results found in rodents, with dose-dependent hyperactivity followed by suppression. This regime also leads to habituation in the behavioral response. The antagonist blocks the increased locomotor activity induced by cannabinoids. Thus, it was observed that there is a similarity in the responses between zebrafish and other mammals, including humans. They also determined the Median Lethal Concentration (LC50) for chronic exposure of zebrafish embryos of THC, 3.37 mg/L (0.01 mM), but concluded that further validation and research of receptor interactions was needed ahead being proved that the zebrafish embryo could be a helpful tool for the preclinical screening cannabinoids.

Achenbach et al. [22] analyzed the uptake, metabolism, and behavioral effects of cannabinoids on zebrafish larvae, comparing the uptake kinetics and metabolism of THC and CBD alone, or in combination with their effects on larval behavior. They proposed that both compounds had distinct behavioral patterns and concentration response profiles. Additionally, the uptake kinetics observed for each compound appeared to correlate with the change in behavior observed in the behavioral assays. When the mix of THC and CBD have been tested, alterations have been noted in each the behavioral activity and the absorption kinetics of each compost when compared when they were tested alone. Again, the results in the zebrafish larvae are like those found in mammalian systems, showing the great potential of this fish in studies with cannabinoids.

In another study, Pandelides et al. [100] proved that the developmental exposure to CBD alters longevity and health span of zebrafish. The fish were exposed during larval development to different concentrations of CBD, ranging from 0.02 to 0.5 μM, for further evaluation of aging in F0 (exposed generation) and in their F1 offspring (two and a half years later). Submitting F0 to CBD, was observed a survival increase (approximately 20%), however, the size (wet weight and length) of female fish was reduced. Even with increased survival, no effects on age-related loss of locomotor function were observed. Regarding fertility, the effects varied by sex and dose. There was a reduction in sperm concentration in males at a dose of 0.5 μM of CBD, while at a dose of 0.1 μM, there was an increase in egg production in females. Analogous to other model systems, the aged control zebrafish exhibited increased kyphosis as well as increased expression markers of senescence and liver inflammation. Exposure to CBD reduced the expression of many of these genes in a dose-dependent manner compared to age-matched controls. CBD effects on size, gene expression and reproduction were not passed on in the F1 generation, suggesting that the influence on aging was not crossed. Although they had been not able to set up the precise mechanism by which CBD caused these effects, a significant alteration in zebrafish development have been observed when CBD was administered. The results of that study highlight that there could be lifelong, sex-dependent outcomes following exposure to CBD during crucial developmental periods.

#### Epilepsy and anxiety

Precursor works have already demonstrated the hypnotic and antiepileptic effects of Cannabidiol since the second half of the last century [101–103], being the results also corroborated with the zebrafish model. Similarly, recent data suggests potential anticonvulsant activity for synthetic cannabinoids, phytocannabinoids, and *C. sativa* e.g., drugs targeting the eCB [103–105]. It was also reported with zebrafish model as shown by Pinder [106], who examined zebrafish behavior in response to 3 mg/L and 7 mg/L of CBD. Behavior was assessed using the novel tank dive behavioral test (NDT). That consists of a test of anxiety displayed by individual zebrafish, and it is linked to predator avoidance behaviors. No significant difference among the treatments was found when compared against control group in anxiety-related bottom-dwelling behavior. However, it was suggested that the CBD impacted the motor activity of the animals, as there was a difference between the groups in terms of speed and time spent on movement. Also concluding the importance of future studies that should use behavioral assays that measure different anxiety-related behaviors, such as the shoaling test that assesses social anxiety.

Prasad et al. [107] also demonstrated that anxiety levels in zebrafish could be measured using NDT. Like humans, male and female zebrafish differed in hormonal composition and therefore responded to treatments differently. In their study, zebrafish were treated with CB receptor agonists anandamide and WIN 55,212‐2 and CB2 inverse agonist JTE‐907 to model the extent the eCB system influenced anxiety. The decreased amount of time spent in the upper zone by the JTE + Anandamide treated fish, combined with the increased time classified as highly mobile, suggested that JTE‐907 had an anxiogenic effect. Anxiolytic effects were observed the WIN treatment group, particularly in male treated fish, suggesting that male treated fish were more receptive to the anxiolytic effects of a non‐selective CB agonist such as WIN 55, 212. Future studies with a water‐soluble CB1 inverse agonist should offer perception into the differing roles of CB1 and CB2 receptors in cannabis‐based treatments.

#### Teratogenicity and neurotoxicity

Many behavioral studies have also been based on protocols carried out with the zebrafish models. Jensen et al. [19], studying CBD effects on behavior and immune gene expression in zebrafish, concluded that exposure to a 40 mg/L solution of CBD interfered with reduced swimming speed and distance. Moreover, resulting from the immune-related genes studied it was shown that expression of two genes il1b and il17a/f2 were up-regulated and four genes, tgfba, ighm, cd4-1, and s100a10b were significantly down-regulated following CBD treatment.

Stewart and Kalueff [84] studied the behavioral effects of acute THC exposure in adult zebrafish, observing the effects of acute 20 min exposure on the animal’s behavior in the novel tank test. The management of THC at doses of 30 and 50 mg/L produced an anxiogenic-like impairment of top swimming, collectively with a slower and more continuous bottom swimming, showing one more time that the behavioral effects of this compound in zebrafish seem to parallel the respective rodent and human findings. Collectively, this emphasizes the developing importance of novel rising aquatic models in translational drug abuse studies and small molecule screening.

Hasumi et al. [108] found that CBD did not produce a dose-dependent inhibitory effect on locomotor activity in zebrafish, with both 0.5 and 10 μg/mL concentrations reducing the speed and distance of swimming. However, 10 μg/mL CBD was observed to attenuate the responses of larvae exposed to darkness. It was found that CBD and WIN induced temporary locomotive disorders and that drug withdrawal for 24 h resulted in an attenuation of drug-induced low activity. Based on these observations, it was concluded that assessing symptoms during and after drug exposure was a valid method for investigating pharmacological effects in a fish model, having these findings important implications with respect to the persistence of drug-associated complications.

Carty et al. [87], also studying the developmental effects of CBD and THC in zebrafish, exposed the fish from blastula through larval stage (96 hpf) to 0.3, 0.6, 1.25, 2.5, 5 mg/L (1, 2, 4, 8, 16 mM) of THC or 0.07, 0.1, 0.3, 0.6, 1.25 mg/L (0.25, 0.5, 1, 2, 4 mM) of CBD. Even with the similarity of THC and CBD morphological anomalies, that is edemas, curved axis, eye/snout/jaw/trunk/fin deformities, swim bladder distention, and behavioral abnormalities, the LC50 for THC (3.65 mg/L) was about 7 higher than CBD (0.53 mg/L). After 96 hpf, c-fos (fosab), dazl (deleted in azoospermia like), and vasa (ddx4) were differentially expressed following THC exposure, however only c-fos expression have been increased by CBD. CBD was more bioconcentrated compared with THC despite higher THC water concentrations. That work supported the potential for persistent developmental impacts of cannabinoid exposure, but more studies are needed to assess latent effects and their molecular mechanisms of toxicity.

Initially, Carty et al. [87] hypothesized CBD would be the least toxic cannabis constituent compared with THC. That was primarily due to its non-psychotropic properties and weak CB1 affinity. Moreover, CBD reflected THC developmental and behavioral toxicities at strikingly lower concentrations. Furthermore, CBD bioconcentrated greater effectively than THC regardless of its lower log P.

In acute assays in zebrafish, THC demonstrated a biphasic response, expanding hyperactivity at a dose of 0.6 mg/L, followed by a suppression of activity dose-dependently at 1.2, 2.4, and 3.4 mg/L. In a chronic assay, zebrafish larvae demonstrated hyperactivity with doses above 1.2 mg/L [85]. These results were consistent with rodents, which reported a stimulation in locomotor activity by THC at low concentrations and suppression at higher concentrations [109].

Regarding teratogenicity or neurotoxicity, Valim Brigante et al. [110] proved that CBD did not induce in exposed zebrafish embryos. No malformations, such as coagulation, tail not detached, malformation of somite, no heartbeat, development of eyes, spontaneous movement, pigmentation, edemas, malformation of head, tail and otoliths, scoliosis, deformity of yolk sac, retarded growth, was observed in morphological analysis of embryos exposed to all tested concentrations of CBD. Although, twenty per cent of embryos exposed to maximal dose of CBD (300 µg/L) hatched after 96hpf, despite the embryos in control solution had already hatched on this period. Embryos submitted to CBD did not presented alteration in acetylcholinesterase activity, however embryos submitted to CBD to 300 µg/L had been from 1.4 up to 1.7-fold more active when compared to the control treatment. Nevertheless, the motor activity of the animals returned to the 48 hpf control levels. These results suggest that the effects observed after CBD exposure are intimately related to CB1 receptor that is present in zebrafish since early stages of development, showing early light effects induced by CBD exposure in concentrations that did not alter biochemical activities. Zebrafish embryos submitted to CBD at concentrations correlated to CBD levels in human plasma confirmed to be modulated via way of means of mechanisms concerning cannabinoids receptors extraordinarily conserved amongst mammals and zebrafish. In zebrafish embryos, CBD modulates motor activity and delays their incubation time, these two events being linked to CB1.

#### Embryogenesis (gastrulation phase)

Many studies with cannabinoids have also been carried out during the zebrafish gastrulation phase [111]. These studies are important because many pregnant women end up using Cannabis during pregnancy, and through zebrafish it is possible to see the results in real time. In zebrafish, gastrulation occurred from 5.25 hpf to 10.75 hpf. At that stage, ectoderm, mesoderm, and endoderm were formed, and primary neurons, including Mauthner cells (M-cells) appear. M-cell neurons first appear around 8–9 hpf in the middle of the developmental period known as gastrulation [112, 113].

Amin et al. [114], exposed the embryos to THC and observed that there was a change in the M-Cell development in zebrafish embryos. The M- cells formed during gastrulation, thus allowing them to examine neuronal morphology of neurons born during the time of exposure. Zebrafish submitted to THC during gastrulation exhibited decreased activity compared with control groups, and presented subtle alterations in M-cell axon diameter and small changes in escape response dynamics to touch, indicating animals exposed to THC during the gastrula phase exhibit small changes in neuronal and muscle morphology that may alter behavior and locomotion.

Ahmed et al. [86], in addition to THC, also tested CBD, submitting fish for 5 h during the gastrulation, with different concentrations of THC, varying from 2 to 10 mg/L, and CBD (varying from 1 to 4 mg/L) to analyze the development. In that study, physical abnormalities at the time of hatching, changes in motor neuron branching and reduced C-start escape responses were observed in zebrafish embryos exposed to THC and CBD for 5 h during gastrulation when compared to the control treatment. The most significant findings resulting from embryos treated with THC and/or CBD exhibited: (i) shorter body lengths and mild deformities, (ii) reduced survival, (iii) reduced heart rates (up to 50% reduction), (iv) decreased frequency of mEPC activity at the NMJ, (v) alterations in branching patterns of secondary MNs, (vi) changes in the expression of postsynaptic nAChRs associated with skeletal musculature and (vii) reduced response rates to sound stimuli. Thus, the results suggest that exposure to THC and CBD very early in life may alter embryonic development.

These studies suggest that a brief exposure to the compound may have an impact on embryonic health and development. In spite that, it would depend on the dose used, therefore, studies with a wider range of doses should be executed. In humans, epidemiological and clinical studies associate maternal cannabis exposure to behavioral disturbances in the offspring linked to increased risk for neuropsychiatric disorders [115]. In rats, maternal exposure of THC changed a series of behaviors in the offspring, including water-induced grooming, increased light sensitivity, and altered exploratory behavior [116]. Recently, the negative impacts of cannabis have expanded and include the non-psychotropic CBD, which disrupts motor-neuron development in zebrafish [86]. This study contrasts with reports that suggest positive health benefits of CBD, such as treating nauseas during pregnancy [117, 118].

#### Knockout models

Another interesting point is that the zebrafish model allows experiments with a range of genetically altered models, consisting of mutations for the research of numerous kinds of diseases. Samarut et al. [119], studied single and synergistic effects of CBD and THC on zebrafish models of neuro-hyperactivity with two previously developed zebrafish models of neuro-hyperactivity. The first one a chemically induced pentylenetetrazol model and the second one a genetic model caused by loss-of-function mutations in the GABA receptor subunit alpha 1. Results showed that both CBD and THC have a significant effect on the behavioral changes induced by both models, providing a validation of the two zebrafish models and sets a platform for future work with cannabinoids, meanly in the context of neuro-hyperactivity disorders.

Serra et al. [120] used a knockout model of zebrafish embryos to study the effect of CBD on *Tuberous sclerosis* complex, a rare disorder caused by mutations in the TSC1 or TSC2 genes, described by generalized tumor growth, intractable epilepsy, cognitive deficits, and autistic behavior. At the end of the study, the authors proposed that CBD selectively modulates levels of phosphorylated rpS6 in the brain, promoting an anxiolytic effect. Zebrafish model of TSC with a TSC2 nonsense mutation (tsc2 ±) was previously described [121, 122].

Griffin et al. [26] used a mutant version of zebrafish (homozygous scn1lab) to research the Dravet's syndrome, classed as a catastrophic early life epilepsy, and characterized by impairment, severe seizures, and increased risk for sudden unexplained death in epilepsy. Yet, refractory to standard antiepileptic drugs, rising preclinical and medical proof indicates that modulation of the endocannabinoid device may be therapeutic in those patients. The study identified through the zebrafish model, that synthetic cannabinoid compounds have anticonvulsant activity.

As seen, several experiments show similarities in the results of studies with the different components of the Cannabis when comparing the zebrafish model with other animal models, making this animal model an alternative to performing screening tests before carrying out tests on mammals, and offering several possibilities through the use of mutant models [22, 83–85].

As observed in the presented studies, a wide range of concentrations were tested with the different cannabinoids, and often, depending on the doses, the effects can be antagonistic, therefore, further studies are needed to elucidate therapeutic doses for the different types of treatment. Thus, both zebrafish and cannabis studies have been increasing exponentially in the last few years indicating that both subjects are extremely important in the face of the challenges of this new century.

As already mentioned, zebrafish experimental models are highly relevant to the human species, therefore, understanding the routes of action of cannabinoids can also contribute to dose modulation studies and therapeutic protocols.

## Conclusions

Different doses of the different components of Cannabis must be studied, as there is still much to be elucidated on how eCB interact. The zebrafish model offers this opportunity for studies to be conducted in less time and at lower costs. This model proves to be effective in elucidating eCB system and also immunological and toxicological responses to various drugs, including Cannabis and its components, and therefore has great potential and extreme relevance for research in the pharmacological area. Besides lowering the experiments’ fee and time, it respects the precept of 3Rs and has benefits over mammalian models such as high prolificacy, transparency of embryos and larvae, external fertilization, rapid development, in addition to high genetic similarity with humans and other mammals. In accordance with international ethical regulations, this model is also accepted as an alternative to in vivo tests on animals, as they are considered in vitro when used in tests for up to five days after fertilization. However, a disadvantage of this model would the lack of current supporting legislation, therefore, governmental and non-governmental entities, both public and private, must make an effort to draw up applicable standards in the context of the use of this animal model.

## Data Availability

See “Materials and methods” section.

## Abbreviations

ADME: Absorption, metabolism and excretion
CBD: Cannabidiol
Dpf: Day post-fertilization
eCB: Endocannabinoid system
LC50: Median Lethal Concentration
NDT: Tank dive behavioral test
THC: ∆9-Tetrahydrocannabinol
